# Supplementary material for: Persistently Increased Expression of PKMzeta and Unbiased Gene Expression Profiles Identify Hippocampal Molecular Traces of a Long‐Term Active Place Avoidance Memory and “Shadow” Proteins
Source: Adv Sci (Weinh). 2026 Mar 13;13(28):e21254. doi: 10.1002/advs.202521254 (PMC13185853; doi:10.1002/advs.202521254)
Supplement: Supplementary file 1 — Supporting File: advs74629‐sup‐0001‐SuppMat.pdf [file ADVS-13-e21254-s001.pdf]

Persistently increased expression of PKMζ and unbiased gene expression profiles identify hippocampal molecular traces of a long-term active place avoidance memory and 'shadow' proteins

Jiyeon Han, Alejandro Grau-Perales, Rayna M. Harris, Edith Lesburgueres, Hsin-Yi Kao, Asit Pal, Juan Marcos Alarcon, Todd C. Sacktor, Stefano Martiniani, Hans A. Hofmann*, Andre A. Fenton**

SUPPLEMENTARY INFORMATION

Table S1: Behavioral end-point measures of place avoidance.

Table S2: Discovery-driven analysis of GO terms that are positively correlated with $PC1_{mem}$.

Table S3: Analysis of GO terms hypothesized to play a role in learning and memory.

Figure S1. PKMζ puncta in dendrites and spines of memory-tagged CA1 pyramidal cell dendrites.

Figure S2. Quality control of RNA-sequencing data.

Figure S3. Experiment 2 number of datasets.

Figure S4. Astrocytic genes.

Figure S5. Robust detection of IEGs and DEGs that predict whether the sample was from the trained or control groups of mice using three non-linear methods.

Figure S6. Comparing the performance of Pearson, Spearman, and Xi correlations to analysis in detecting linear and non-linear co-expression relationships.

Figure S7. Impact of Correlation Methods and WGCNA Parameters on Module Detection

Figure S8. Gene Ontology analysis of the genes co-assigned with the three key genes by robust Louvain community detection.

Figure S9. Markov clustering (MCL) of yoked and trained CAP matrices by inflation parameter.

Figure S10. Representative final subcluster with mixed positive and negative correlations.

Figure S11. PCA of the z-scored raw expression matrix fails to separate candidate genes or training conditions.

Figure S12. PCA visualization of raw Sigma(Σ) scores and TOM similarity.

Figure S13. Two-dimensional scatter plots of selected metric pairs.

Figure S14. Robustness of t-SNE and UMAP Dimensionality Reductions Across Parameter Settings.

Figure S15. Ablation study using different gene-gene correlation measurements.

Variable	End-point Measure	Meaning
**TotalPath**	**Total Path**	**Total distance walked by the animal, in meters.**
**NumEntrances**	**Number of Entrances**	**Total number of times the animal entered the shock zone.**
**Time1stEntr**	**Time to first entrance**	**Amount of time it took the animal to enter the shock zone for the first time**
**Time2ndEntr**	**Time to second entrance**	**Amount of time it took the animal to enter the shock zone for the second time**
Time3rdEntr	Time to third entrance	Amount of time it took the animal to enter the shock zone for the third time
Time4thEntr	Time to fourth entrance	Amount of time it took the animal to enter the shock zone for the fourth time
Time5thEntr	Time to fifth entrance	Amount of time it took the animal to enter the shock zone for the fifth time
**Path1stEntr**	**Path to first entrance**	**Distance the animal walked before entering the shock zone for the first time**
**Path2ndEntr**	**Path to second entrance**	**Distance the animal walked before entering the shock zone for the second time**
Path3rdEntr	Path to third entrance	Distance the animal walked before entering the shock zone for the third time
Path4thEntr	Path to fourth entrance	Distance the animal walked before entering the shock zone for the fourth time
Path5thEntr	Path to fifth entrance	Distance the animal walked before entering the shock zone for the fifth time
**Speed1stEntr**	**Speed to first entrance**	**Speed at which the animal entered the shock zone for the first time**
**Speed2ndEntr**	**Speed to second entrance**	**Speed at which the animal entered the shock zone the second time**
**EntrPerDist**	**Entrances per distance**	**Number of entrances normalized by the total distance walked (#Ent/Tpath)**
**NumShock**	**Number of shocks**	**Total amount of shocks the animal received**
Timeto1stS	Time to first shock	Amount of time it took the animal to get shocked for the first time
Pathto1stS	Path to first shock	Distance the animal walked before getting shocked for the first time
**Speed**	**Speed**	**Average speed of the animal throughout the session**
**sdSpeed**	**SD of speed**	**Standard deviation of Speed**
**Linearity**	**Linearity**	**How straight or tortuous was the walking of the animal**
**MaxTimeAvoid**	**Maximum time avoided**	**Maximum amount of time that the animal stood away from the shock zone**
**MaxPathAvoid**	**Maximum path avoided**	**Maximum distance walked without entering the shock zone**
**TimeShockZone**	**Time in shock zone**	**Total time spent in the shock zone**
**pTimeShockZone**	**proportion in shock zone**	**Proportion of time spent in the quadrant where the shock zone is**
**pTimeCCW**	**Proportion in ccw**	**Proportion of time spent in the quadrant counterclockwise from the target quadrant**
**pTimeOPP**	**Proportion in opp**	**Proportion of time spent in the quadrant opposite from the target quadrant**
**pTimeCW**	**Proportion in cw**	**Proportion of time spent in the quadrant clockwise from the target quadrant**
**RayleigLength**	**Rayleigh length (A)**	**Length of the vector describing where the animal spent most of its time (the longer the vector, the stronger the preference for that spot. It ranges 0-1**
**RayleigAngle**	**Rayleigh angle (A)**	**Angle of the vector describing where the animal spent most of its time. It ranges 0-360.**
Polavg	Polar average (B)	Average degree where the animal spent most of time. It ranges 0-360.
PolSd	Polar SD	Standard deviation of polar average
PolMin	Polar minimum (C)	Proportion of time spent in the least preferred 10-degree bin
PolMinBin	Polar minimum bin (C)	The least preferred 10-degree bin
**Min50%RngLoBin**	**Minimum 50% low bin (D)**	**One of the boundaries that flank the least preferred arc (combination of least preferred bins)**
Min50HiBin	Minimum 50% high bin (E)	The other boundary that flanks the least preferred arc
PolMax	Polar maximum (F)	Proportion of time spent in the most preferred 10-degree bin
PolMaxBin	Polar maximum bin (F)	Most preferred bin
Max50LoBin	Maximum 50% low bin (G)	One of the boundaries that flank the most preferred arc (combination of most preferred bins)
Max50HiBin	Maximum 50% high bin(H)	The other boundary that flanks the most preferred arc
AnnMin	Annular minimum (I)	Proportion of time spent in the least preferred concentric circle
AnnMinBin	Annular minimum bin (J)	The least preferred concentric circle
AnnMax	Annular maximum (K)	Proportion of time spent in the most preferred concentric circle
AnnMaxBin	Annular maximum bin (L)	The most preferred concentric circle
Ann	Annular Average	Average annulus where the animal spent the time
AnnSD	Annular SD	Standard deviation of Annular average
**AnnularSkewnes**	**Annular Skewness**	**Skewness of the annular (histogram) distribution**
**AnnularKurtosis**	**Annular Kurtosis**	**Kurtosis of the annular (histogram) distribution**
**ShockPerEntrance**	**Shock per entrance**	**The number of shocks per entrance**

Table S1: Behavioral end-point measures of place avoidance. The subset of measures used to quantify place avoidance are indicated by bold. The full set of raw behavioral data are available here: BehaviorRawData. The $PC1_{mem}$ and $PC2_{act}$ retention scores for each mouse are available here: BehaviorPCAdata.

Domain	Name	Total	Present	Genes
MF	transcription regulator activity	1789	50	Npas4, Smad7, Fosl2, Nfil3, Btg2, Egr4, Srf, Ahr, Junb, Nr4a2, Atf3, Jun, Zfp281, Cited2, Nr4a3, Mtdh, Zbtb33, Egr1, Foxo1, Plagl1, Abhd2, Fosb, Rora, Dnajb1, Jund, Zfp869, Nr4a1, Zfp275, Klf2, Rfx2, Hspa1b, Maml2, Zfp655, Fos, Foxg1, Egr3, Ski, Jmjd1c, Erf, Bach1, Arid5b, Irf2bp2, Sox9, Jdp2, Pou3f3, Gmeb2, Myc, Med7, Kdm7a, Klf7
MF	MAP kinase tyrosine/serine/threonine phosphatase activity	13	6	Dusp8, Dusp4, Dusp16, Dusp6, Dusp14, Dusp5
MF	protein tyrosine/threonine phosphatase activity	10	5	Dusp8, Dusp4, Dusp16, Dusp6, Dusp5
MF	molecular function regulator activity	2135	39	Rgs2, Ccnk, Smad7, Fbxw7, Sgk1, Bdnf, Scg2, Ranbp2, Eif5, Hmgcr, Irs2, Jun, Cited2, Rgs4, Flrt3, Ppp1r15a, Arpp19, Dnaja1, Apaf1, Rasl11a, Ccno, Dnajb4, Hsph1, Dnajb1, Jag1, Hspa1b, Calu, Ppp1r15b, Kitl, Ing2, Sh2d3c, Hsp90aa1, Rap2a, Il16, Cdkn1a, Arhgef3, Bag3, Tgfb3, Cpeb2
MF	myosin phosphatase activity	73	6	Dusp8, Dusp4, Dusp16, Dusp6, Dusp14, Dusp5
BP	learning or memory	326	20	Npas4, Ptgs2, Plk2, Arc, Nptx2, Adrb1, Pak6, Sgk1, Bdnf, Btg2, Kcnk10, Syt4, Srf, Nr4a2, Hmgcr, Jun, Egr1, Fos, Nedd9, Ntrk2
BP	rhythmic process	353	16	Adrb1, Fbxw7, Nfil3, Bdnf, Ahr, Homer1, Per1, Jun, Fzd4, Adamts1, Egr1, Rora, Siah2, Ciart, Per2, Ntrk2
BP	trans-synaptic signaling by neuropeptide	3	3	Bdnf, Syt4, Ntrk2
BP	response to heat	93	8	Ptgs2, Arpp21, Dnaja4, Dnaja1, Dnajb1, Hspa1b, Hsp90aa1, Bag3
BP	glucose metabolic process	224	11	Errfi1, Ranbp2, Irs1, Irs2, Atf3, Arpp19, Foxo1, Rora, Dyrk2, Serp1, Myc
BP	leukocyte activation	1033	24	Tnip2, Smad7, Fosl2, Peli1, Fzd5, Nfil3, Cxadr, Srf, Ahr, Irs2, Jun, Nr4a3, Egr1, Hsph1, Rora, Jund, Kitl, Egr3, Hsp90aa1, Cdkn1a, Irf2bp2, Arid1a, Myc, Nedd9
BP	trans-synaptic signaling by BDNF, modulating synaptic transmission	5	3	Bdnf, Syt4, Ntrk2
BP	cell migration involved in sprouting angiogenesis	55	6	Ptgs2, Plk2, Fbxw7, Srf, Nr4a1, Egr3
BP	nuclear receptor-mediated glucocorticoid signaling pathway	16	4	Bdnf, Per1, Arid1a, Ntrk2
BP	regulation of type B pancreatic cell proliferation	17	4	Errfi1, Irs2, Nr4a3, Nr4a1
BP	response to amphetamine	38	5	Rgs2, Ranbp2, Nr4a2, Rgs4, Nr4a1
BP	female pregnancy	235	10	Errfi1, Ptgs2, Rgs2, Junb, Cited2, Fosb, Fos, Arid1a, Slc2a1, Tgfb3
BP	regulation of cell junction assembly	236	10	Fzd5, Bdnf, Flrt3, Lrrtm2, Amigo2, Fermt2, Rap2a, Slitrk5, Ntrk2, Vcl
BP	regulation of protein transport	521	15	Slc16a1, Ptgs2, Syt4, Hmgcr, Irs1, Irs2, Dnaja1, Foxo1, Plk3, Cyp51, Hsp90aa1, Serp1, Klf7, Bag3, Tgfb3
BP	organic anion transport	460	14	Slc16a1, Ptgs2, Rgs2, Slc2a3, Bdnf, Syt4, Eprs, Irs2, Slc25a25, Rgs4, Slc6a6, Myc, Slc2a1, Ntrk2
BP	vascular process in circulatory system	238	10	Ptgs2, Rgs2, Adrb1, Hmgcr, Klf2, C2cd4b, Gch1, Fermt2, Slc2a1, Dusp5
CC	transcription factor AP-1 complex	5	4	Junb, Jun, Jund, Fos
CC	cell junction	2266	42	Npas4, Slc16a1, Smad7, Slc2a3, Pcdh8, Arc, Nptx2, Adrb1, Acan, Pak6, Fzd5, Bdnf, Kcnj2, Cxadr, Frmd6, Cul3, Cpeb4, Syt4, Eif5, Homer1, Flrt3, Mtdh, Fzd4, Kcna4, Dnajb1, Jag1, Mfap3l, Nr4a1, Lrrtm2, Fermt2, Egr3, Cldn12, Rap2a, Il16, Slitrk5, Slc6a6, Ctnnd1, Slc2a1, Gad1, Nedd9, Ntrk2, Vcl
CC	plasma membrane region	1409	26	Slc16a1, Ptgs2, Slc2a3, Pcdh8, Arc, Nptx2, Kcnj2, Cxadr, Frmd6, Irs1, Flrt3, Mtdh, Kcna4, Jag1, Hspa1b, Lrrtm2, Fermt2, Sh2d3c, Hsp90aa1, Rap2a, Arl13b, Slc6a6, Ctnnd1, Slc2a1, Nedd9, Ntrk2
CC	cell-cell junction	538	14	Smad7, Fzd5, Kcnj2, Cxadr, Frmd6, Flrt3, Mtdh, Fzd4, Jag1, Fermt2, Cldn12, Ctnnd1, Slc2a1, Vcl
CC	neuronal dense core vesicle	34	4	Adrb1, Bdnf, Scg2, Syt4

Table S2: Discovery-driven analysis of GO terms that are positively correlated with PC1. Column 1 lists the three GO domains: Biological Process (BP), Cellular Compartment (CC), and Molecular Function (MF). Columns 2 lists the top 5 most significant functional categories that are represented in the list of 232 genes that are positively correlated with PC1, a measure of avoidance behavior.

	Candidate Genes	DG DEGs	LTP Genes from Sanes & Lichtman 1999
Response to stimulus (GO-0050896)	Fos, Fosl2, Npas4, Grin1, Gria1, Gria2, Pick1, Fmr1, Camk2a, Wwc1, Prkcb, Prkcz, Prkci	2410002F23Rik, Abhd2, Adrb1, Ahr, Ankrd13c, Ankrd27, Apaf1, Arhgef3, Arid1a, Arid5b, Arl13b, Arpp21, Aste1, Atf3, B3gnt2, Bach1, Bag3, Bdnf, Btg1, Btg2, C2cd4b, Ccar2, Ccnk, Cdkn1a, Chrd, Cited2, Cpeb2, Cpeb4, Ctnnd1, Cul3, Cxadr, Cyp51, Dnaja1, Dnaja4, Dnajb1, Dusp16, Dusp4, Dusp5, Dusp6, Dusp8, Dvl2, Dyrk2, Egr1, Egr3, Errfi1, Fbxl2, Fbxw7, Fermt2, Flrt3, Fos, Fosb, Fosl2, Foxo1, Frmd6, Fzd4, Fzd5, Gadd45a, Gadd45g, Gch1, Gcnt2, Gpr19, Hmgcr, Hmgn2, Homer1, Hsp90aa1, Hspa1a, Hspa1b, Hsph1, Ifrd1, Il16, Ing2, Irs1, Irs2, Jag1, Jun, Junb, Jund, Kcnj2, Kctd12, Kdm6b, Kitl, Klf2, Klf7, Lbh, Lemd3, Lmna, Maml2, Mcl1, Mest, Mn1, Mtdh, Myc, Nedd9, Nfil3, Npas4, Nr4a1, Nr4a2, Nr4a3, Ntrk2, Nuak1, Odc1, Olfml2b, Pak6, Peg10, Peli1, Per1, Per2, Piga, Plk2, Plk3, Ppp1r15a, Ppp1r15b, Ptgs2, Pxn, Ranbp2, Rap2a, Rasd1, Rfx2, Rgmb, Rgs2, Rgs4, Rora, Scg2, Sde2, Sec61b, Serp1, Sgk1, Sh2d3c, Siah2, Ski, Slc16a1, Slc25a25, Slc2a1, Slitrk5, Smad7, Sox9, Srf, Tgfb3, Tiparp, Tnip2, Tra2b, Traip, Tsc22d2, Usp27x, Wdr45b, Zbtb33, Zfand5, Zfp207, Zfp668	Ache, Adcy1, Adcy10, Adcy2, Adcy3, Adcy4, Adcy5, Adcy6, Adcy7, Adcy8, Adcy9, Adra1a, Adra1b, Adra1d, Adra2a, Adra2b, Adra2c, Adrb1, Adrb2, Adrb3, Bdnf, Cacna1a, Cacna1b, Cacna1c, Cacna1d, Cacna1e, Cacna1f, Cacna1s, Calb1, Calm1, Calm2, Calm3, Camk1, Camk4, Capn1, Capn10, Capn2, Capn3, Ccr7, Cd47, Cdh1, Cdh2, Chrm1, Chrm2, Chrm3, Chrm4, Chrm5, Chrna1, Chrna3, Chrna7, Chrnb1, Chrnb2, Chrnb3, Cnr1, Cnr2, Creb1, Dlg4, Drd1, Edn1, Efna5, Egf, Egr1, Egr2, Epha5, Erbb4, Fgf2, Fyn, Gabbr1, Gabra1, Gabra5, Gabrb1, Gabrb2, Gabrb3, Gap43, Gfap, Gria1, Gria2, Grin1, Grin2a, Grin2d, Grm1, Grm4, Grm5, Grm7, Gucy1a2, Gucy1b2, Gucy2c, Gucy2d, Gucy2e, Gucy2g, Homer1, Homer2, Homer3, Htr1a, Htr1b, Htr1f, Htr2a, Htr2b, Htr2c, Htr3a, Htr3b, Htr4, Htr5a, Htr5b, Htr6, Htr7, Il1b, Inhba, Itga1, Itga10, Itga11, Itga2, Itga2b, Itga3, Itga4, Itga5, Itga6, Itga7, Itga8, Itga9, Itgad, Itgae, Itgal, Itgam, Itgav, Itgax, Itgb1, Itgb1bp1, Itgb2, Itgb2l, Itgb3, Itgb4, Itgb5, Itgb6, Itgb7, Itgb8, Itgbl1, Itpka, Itpkb, Itpkc, L1cam, Mapk1, Mapk10, Mapk11, Mapk12, Mapk14, Mapk3, Mapk4, Mapk6, Mapk7, Mapk8, Mapk9, Mas1, Ncam1, Ngf, Nos1, Nos3, Nrg1, Nrg2, Nrg3, Nrgn, Ntrk2, Oprd1, Oprl1, Oprm1, Parp1, Phpt1, Pla2g10, Pla2g1b, Pla2g2a, Pla2g2d, Pla2g2e, Pla2g2f, Pla2g3, Pla2g4a, Pla2g4f, Pla2g5, Pla2g6, Pla2g7, Plat, Plcb1, Plcb2, Plcb3, Plcb4, Plcg1, Plcg2, Plg, Pnoc, Ppp3ca, Ppp3cb, Ppp3cc, Prkacb, Prkar1b, Prkcg, Prkcz, Prkg1, Ptn, Rab3a, Rarb, S100b, Serpine2, Sptbn1, Src, Stx1b, Syp, Th, Thy1, Tnc, Ube3a, Vamp2, Vamp3, Vamp4, Vamp8
Translation (GO-0006412)	Fmr1	Btg2, Cpeb2, Cpeb4, Eif5, Mrps31, Ppp1r15a, Ppp1r15b, Rgs2, Serp1	Itga2, Mapk1, Mapk3
Synapse organization (GO-0050808)	Npas4, Arc, Grin1, Gria1, Pick1, Numb	Amigo2, Arc, Bdnf, Chrd, Flrt3, Fzd5, Homer1, Lrrtm2, Nedd9, Npas4, Ntrk2, Pcdh8, Rap2a, Slitrk5	Ache, Bdnf, Cacna1a, Cacna1s, Camk1, Cd47, Cdh1, Cdh2, Chrna1, Chrna7, Chrnb1, Chrnb2, Dlg4, Drd1, Efna5, Epha5, Erbb4, Fyn, Gabra1, Gabra2, Gabra3, Gabra5, Gabra6, Gabrb2, Gabrb3, Gap43, Gria1, Grin1, Grin2a, Grm5, Homer1, Htr1a, Htr4, Icam5, Itga3, Itgb1, Itgb3, Itpka, L1cam, Mapk14, Nrg1, Nrg2, Nrg3, Ntrk2, Ptn, Rab3a, Syn1, Tnc, Ube3a
Learning or memory (GO-0007611)	Fos, Npas4, Arc, Grin1, Gria1, Prkcz	Adrb1, Arc, Bdnf, Btg2, Chrd, Egr1, Fos, Hmgcr, Jun, Nedd9, Npas4, Nptx2, Nr4a2, Ntrk2, Pak6, Plk2, Ptgs2, Sgk1, Srf, Syt4	Adcy1, Adcy3, Adcy8, Adra1b, Adrb1, Adrb2, Bdnf, Cacna1c, Cacna1e, Calb1, Camk4, Chrna7, Chrnb2, Cnr1, Creb1, Drd1, Egr1, Gabra5, Gabrb3, Gria1, Grin1, Grin2a, Grm4, Grm5, Grm7, Gucy2d, Htr2a, Htr2c, Htr6, Htr7, Il1b, Itga3, Itga5, Itga8, Itgb1, Ncam1, Ngf, Nrg1, Nrgn, Ntrk2, Oprl1, Pla2g6, Plcb1, Prkar1b, Prkcg, Prkcz, Ptn, S100b, Snap25, Th, Ube3a

Table S3: Analysis of GO terms hypothesized to play a role in learning and memory. Column 1 contains four GO categories representing four hypotheses about which genes are important for memory formation and recall: 1) response to stimulus, 2) translation, 3) synapse organization, and 4) learning or memory. Column 2 contains the candidate genes described in the introduction. Column 3 contains genes that were differentially expressed in the dentate gyrus (DG) of trained and yoked control mice a day after memory

training and thirty minutes after unreinforced expression of active place avoidance memory. Of those DEG, 149 are associated with hypothesis 1 that genes would be related to response to stimulus, which includes many immediate early genes that trigger responses in signaling cascades with membrane-bound receptors. Only 9 genes were associated with the hypothesis that translation machinery that executes the response are needed. Only fourteen genes are associated with "synaptic organization" that includes synaptic receptors and channels that express the response. Twenty genes associated with learning or memory at the behavioral level were differentially expressed. **Adrb1, Arc, Bdnf, Btg2, Chrd, Egr1, Fos, Hmgcr, Jun, Nedd9, Npas4, Nptx2, Nr4a2, Ntrk2, Pak6, Plk2, Ptgs2, Sgk1, Srf and Syt4**. All differentially expressed genes in the learning and memory list also overlap with the genes in the response to stimulus list. Column 4 contains the genes that were reviewed by Sanes and Lichtman, 1999 to play a role in long-term potentiation, a cellular model of memory. This list of 249 contains the reviewed genes and their isoforms. Only seven genes (shown in bold: **Adrb1, Bdnf, Egr1, Homer1, Ntrk2, Stmn4, and Vamp1**) from the Sanes and Lichtman list were found to be differentially expressed in the DG.

SUPPLEMENTARY FIGURES

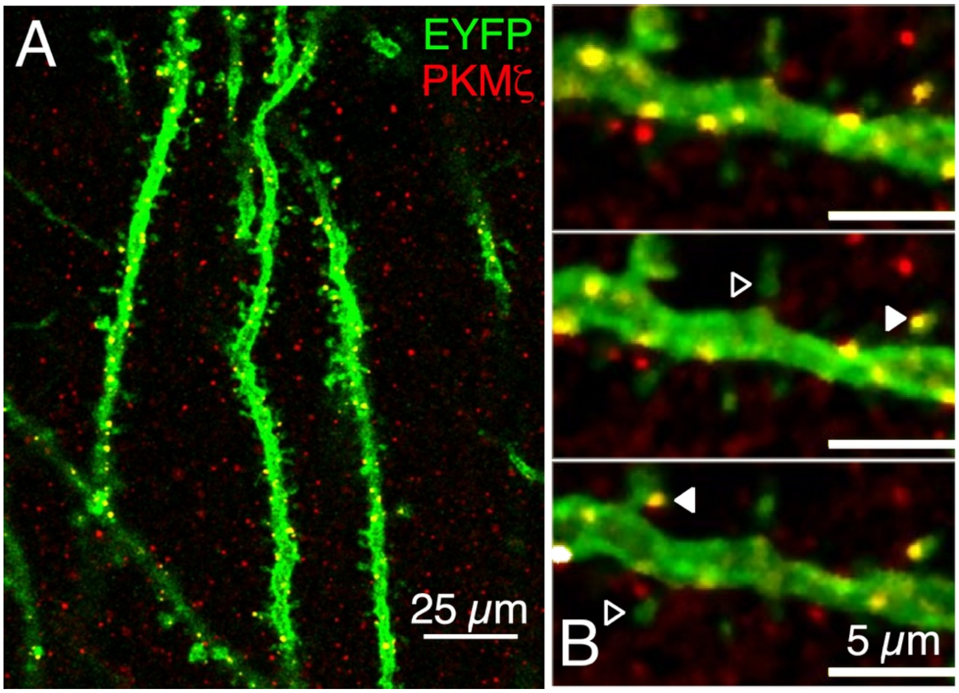

Figure S1. PKMζ puncta in dendrites and spines of memory-tagged CA1 pyramidal cell dendrites.
A) Image of EYFP-tagged CA1 dendrites from tissue prepared for expansion microscopy according to the protocol in ref 72. B) A representative series of three z-stack images with 0.5 μm interval steps. Arrowheads mark examples spine with co-localized PKMζ (closed) and a spine without measurable PKMζ (open).

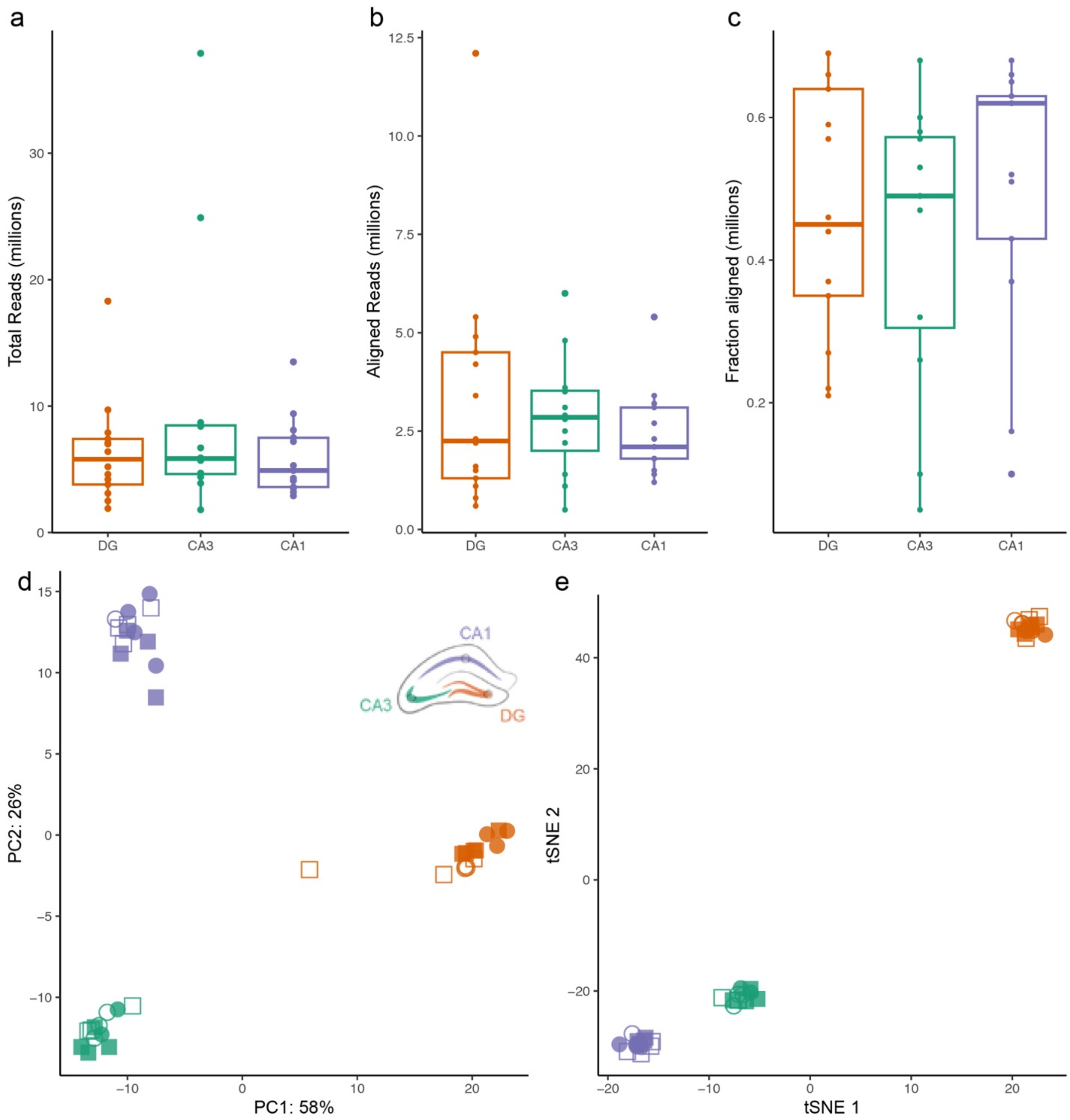

Figure S2. Quality control of RNA-sequencing data. a) On average, each sample yielded 6.9 million raw reads. b) Of those raw reads, on average 2.6 million mapped to the reference transcriptome. c) The alignment efficiency of Kallisto was over 40%. d) Principal component analysis (PCA) and e) t-Distributed Stochastic Neighbor Embedding (t-SNE) both confirm that all samples can be divided into three clusters based on subfield (DG, CA3, or CA1), as expected, with CA1 and CA3 being more similar to one another than to DG.

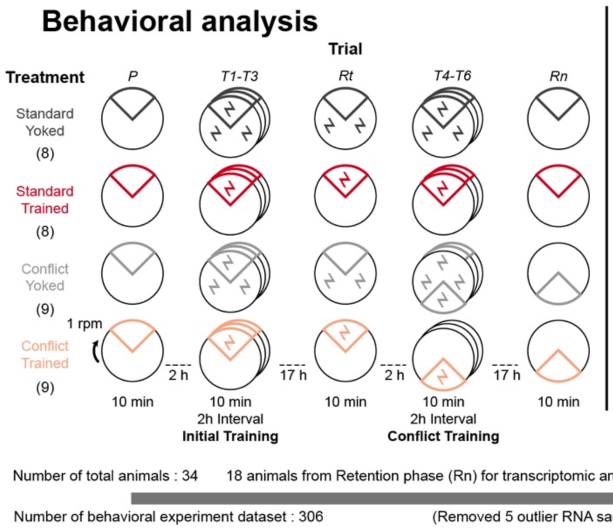

RNA sequencing

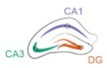

	Animal Count	DG	CA3	CA1	Sequenced Samples
Standard Yoked	4	3	3	1	7
Standard Trained	4	3	2	4	9
Conflict Yoked	5	3	3	4	10
Conflict Trained	5	5	4	4	13
**Total**	**18**	**14**	**12**	**13**	**39**

Figure S3. Experiment 2 number of datasets.

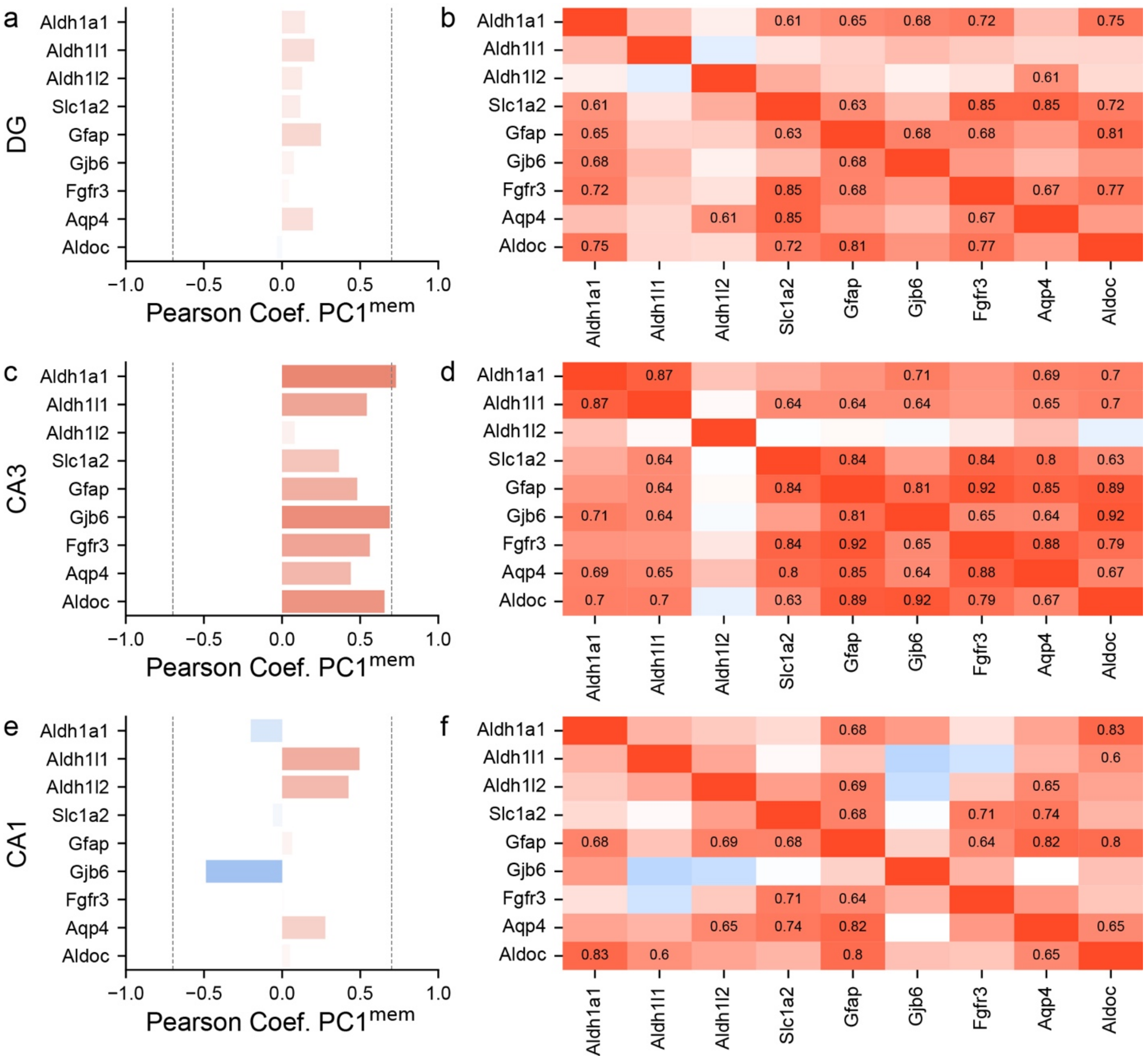

Figure S4. Astrocytic genes. Region-specific candidate gene analysis of astrocyte-related genes. None of the astrocyte-related genes were significantly correlated in DG (a) or CA1 (e), but *Aldh1l1, Aldoc, Fgfr*, and *Gfap* expression in the CA3 all show a correlation coefficient greater than 0.6 to behavioral PC1 (c). These candidate genes are highly, positively correlated with one another, especially in the CA3 (d) and to a lesser extent in the DG (b) and CA1(f).

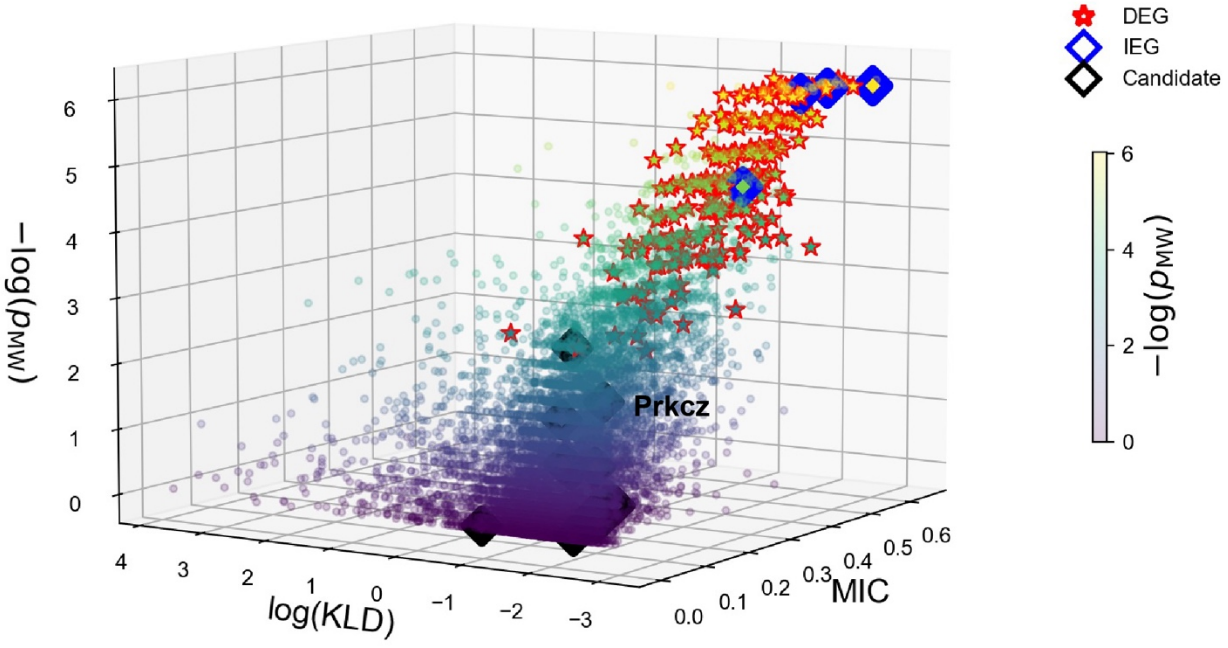

Figure S5. Robust detection of IEGs and DEGs that predict whether the sample was from the trained or control groups of mice using three non-linear methods. X: Kullback-Liebler Divergence (*KLD*) measured in bits. Y: Mutual Information (*MIC*) measured in bits. Z: Probability of the Mann-Whitney statistic (p_{mw}).

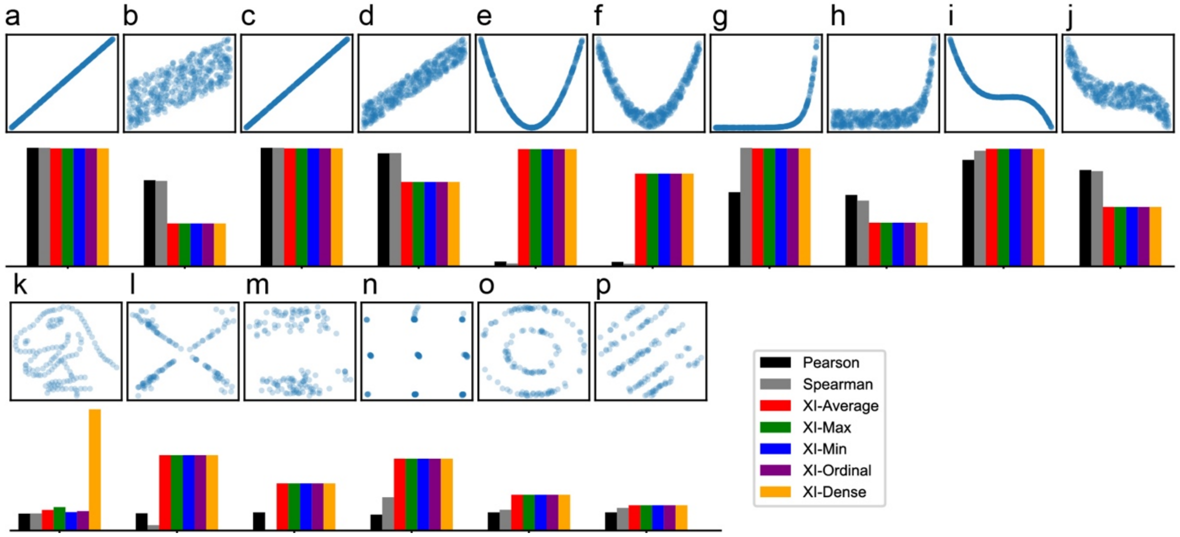

Figure S6. Comparing the performance of Pearson, Spearman, and Xi correlations to analysis in detecting linear and non-linear co-expression relationships.

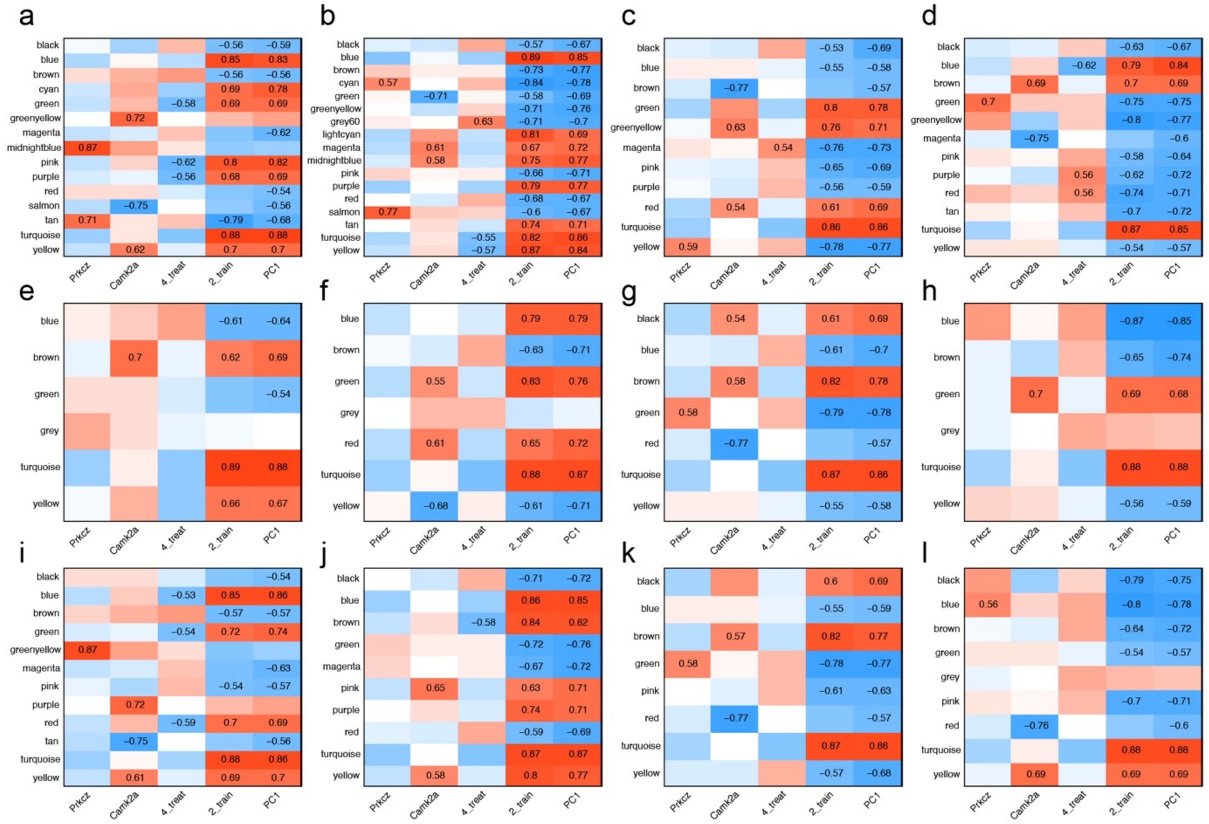

Figure S7. Impact of Correlation Methods and WGCNA Parameters on Module Detection. WGCNA was performed on 712 genes (excluding Prkcz and Camk2a to preserve them as external traits) using four gene–gene correlation matrices: Pearson (a, e, i), xi-correlation (b, f, j), sigma score (c, g, k), and max(Spearman, xi) (d, h, l). Module–trait relationships were evaluated against Prkcz expression, Camk2a expression, four treatment conditions, training status (yoked vs. trained), and $PC1_{mem}$. To assess parameter sensitivity, we varied deepSplit and minModuleSize across three settings: (a–d) deepSplit = 4, minModuleSize = 15, (e–h) deepSplit = 4, minModuleSize = 30, and (i–l) deepSplit = 2, minModuleSize = 15. These dramatic changes in module number and composition make it difficult to perform robust comparisons among the various gene–gene correlation methods.

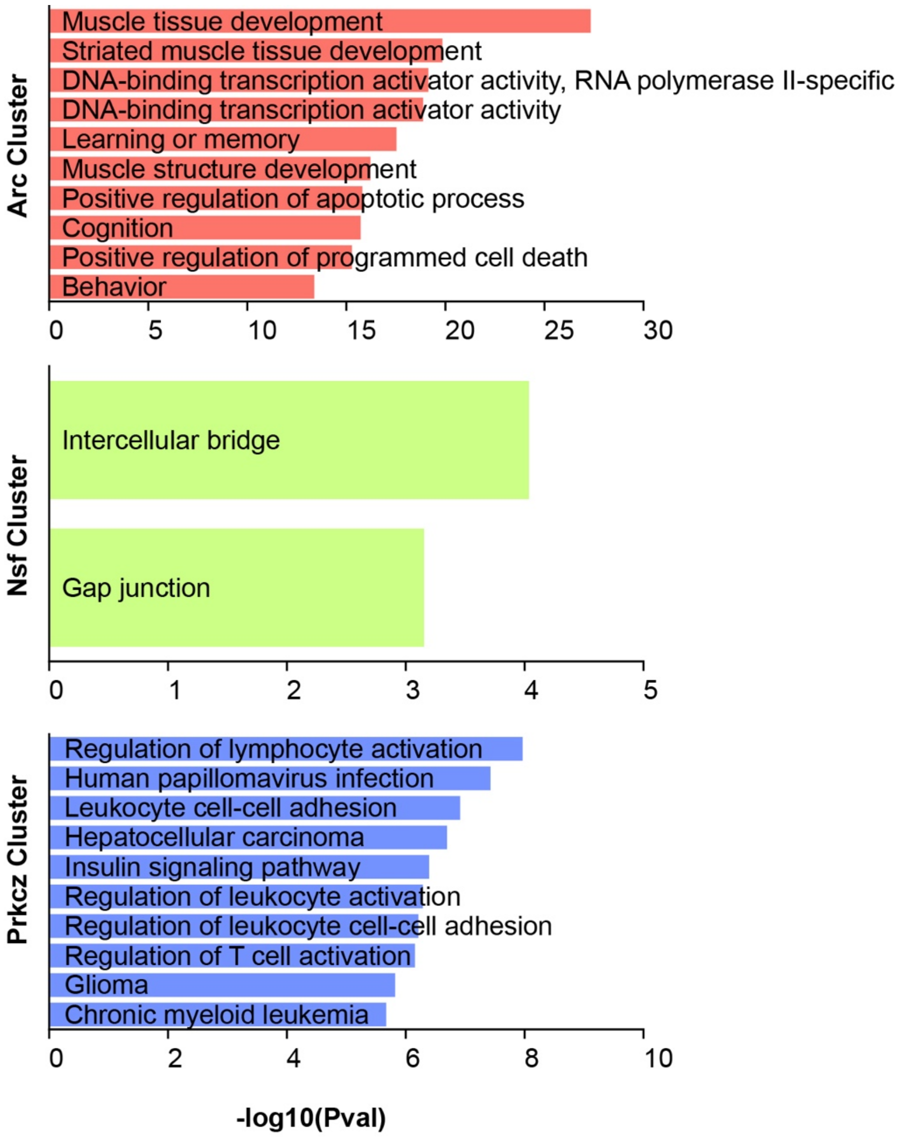

Figure S8. Gene Ontology analysis of the genes co-assigned with the three key genes by robust Louvain community detection.

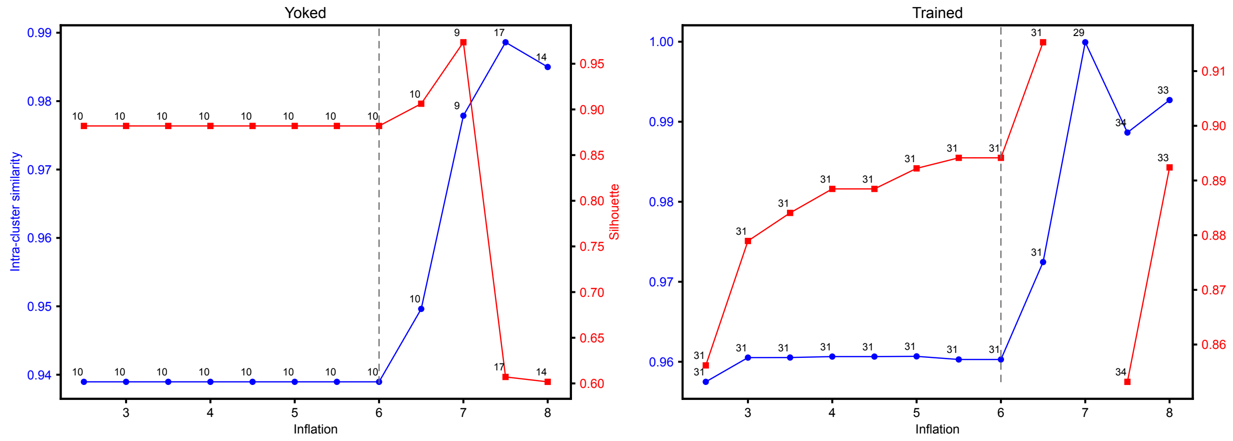

Figure S9. Markov clustering (MCL) of yoked and trained CAP matrices across different inflation parameters. The inflation value determines the granularity of the subclusters. The x-axis represents the tested inflation values. The blue line indicates the average intra-cluster similarity for each clustering result, calculated using the cosine similarity of the yoked and trained CAP matrices. The red line shows the silhouette score computed using the same similarity matrix as the distance metric. The number next to each dot indicates the final number of clusters obtained after MCL clustering. We selected an inflation value of 6, which yielded a stable number of clusters in both yoked and trained conditions, with high scores for both metrics.

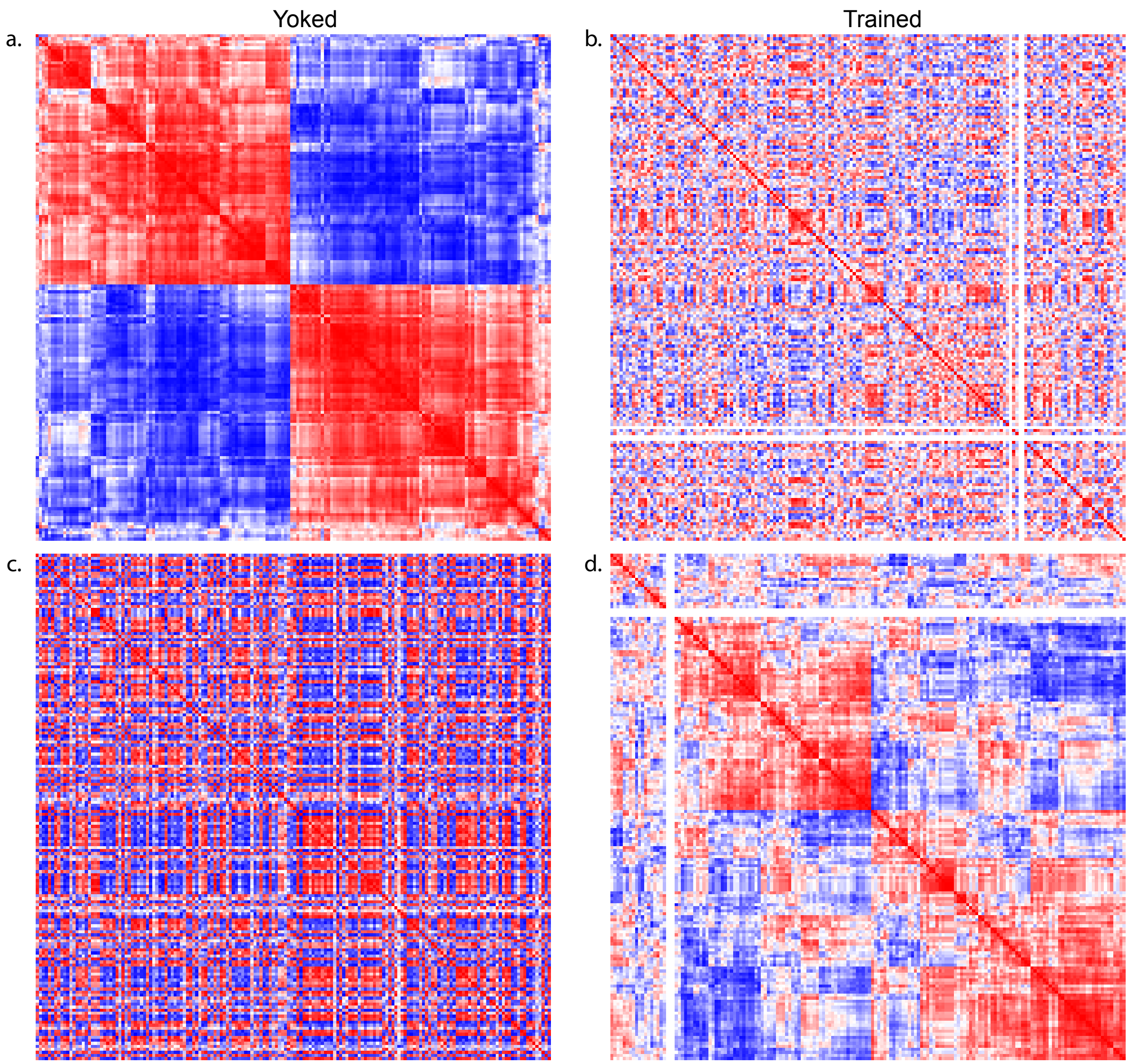

Figure S10. Representative final subcluster with mixed positive and negative correlations. This example uses 168 genes from the Yoked 0 (Y0) cluster. Each heatmap shows the pairwise Pearson correlation values across the raw expression data from yoked and trained animals, ranging from –1 (blue) to 1 (red). The top row (a and b) follows the clustering order of the yoked samples using all 168 × 168 gene pairs, while the bottom row (c and d) follows the clustering order of the trained samples accordingly. Because this figure is based on the yoked sample–derived clustering result (Y0), a clear contrast in color is observed in panel a compared to panel d.

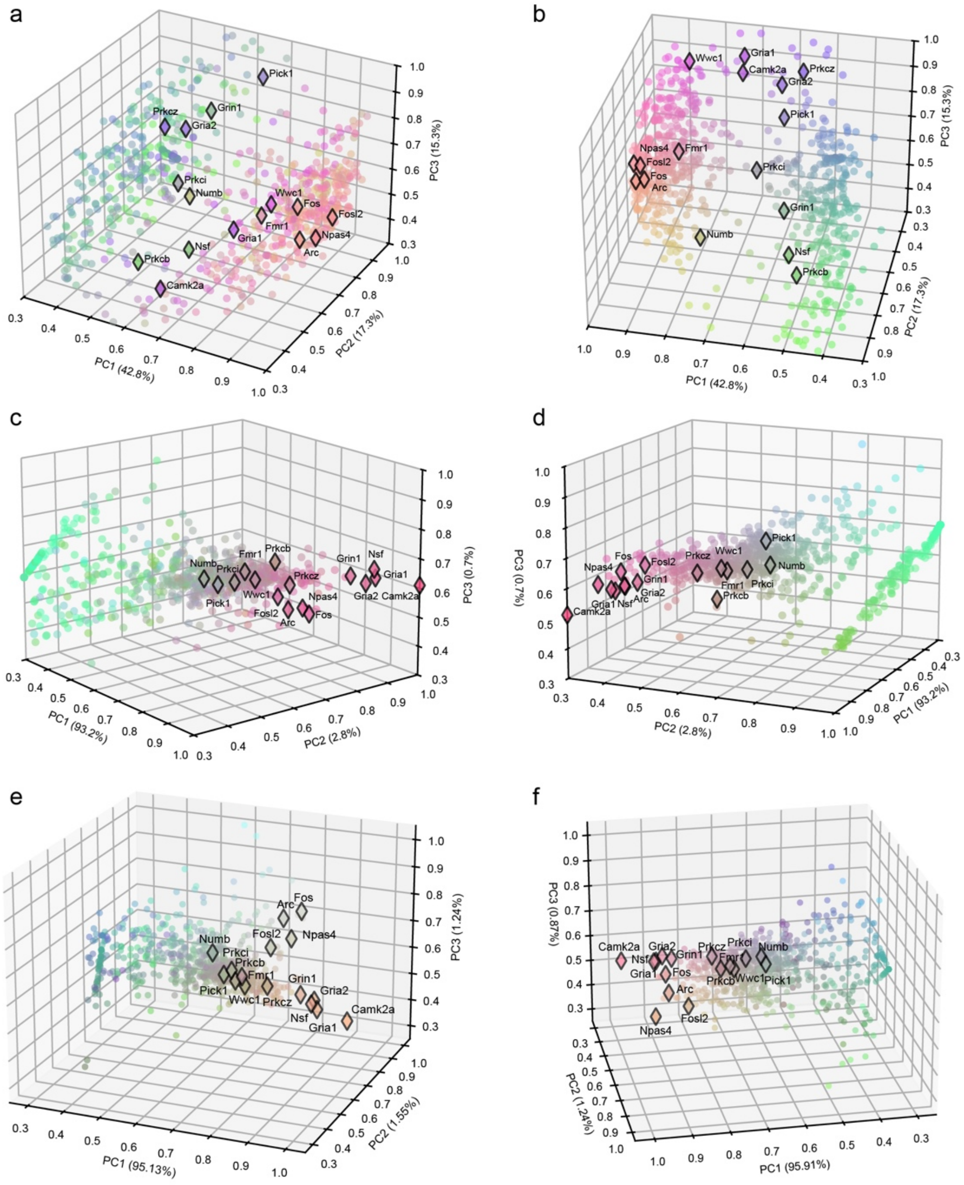

Figure S11. PCA of the z-scored raw expression matrix fails to separate candidate genes or training conditions. PCA projections illustrate the global gene expression profiles of Control (a, c, e) and Trained (b, d, f) samples. Panels a-d represent projections onto common PC space to facilitate direct comparison. a and b show projections after gene-wise Z-scoring which standardizes each gene's variance across samples to emphasize relative expression shifts. c and d present sample-wise Z-scoring projection, which standardize within each sample preserving expression hierarchy. e and f show independent PCA results of sample-wise Z-scoring for each condition. While backbone of the gene network is conserved, it is difficult to distinguish the two conditions using linear dimensionality reduction. Candidate genes are also not clearly separated according to the functions and conditions.

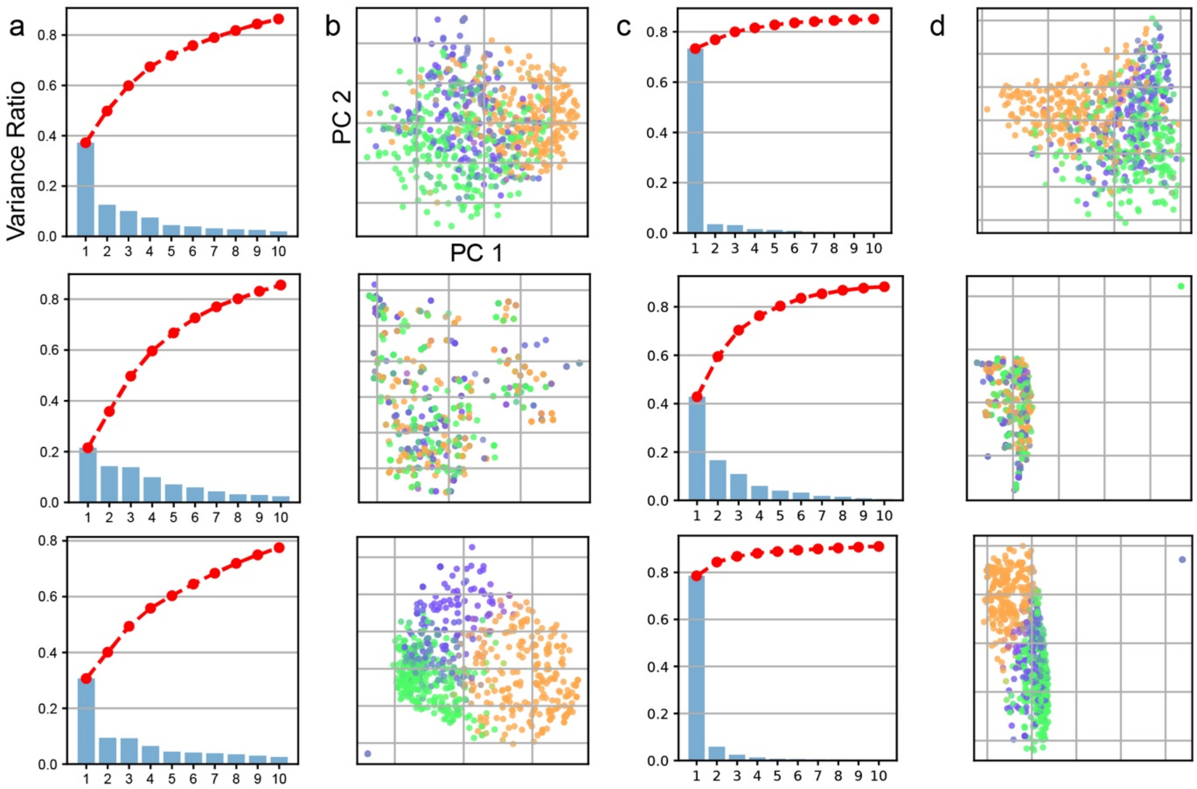

Figure S12. PCA visualization of raw Sigma(Σ) scores and TOM similarity. To compare with our gene–gene co-clustering score approach, we performed PCA using both the raw sigma correlation matrix (a, b) and the Topological Overlap Matrix (TOM) used in WGCNA (c, d). From top to bottom, each row represents all samples combined, yoked samples only, and trained samples only. We applied the same RGB color map as used for the main training clusters. These approaches tended to produce compact or ball-shaped clusters that made it less straightforward to distinguish condition-specific changes.

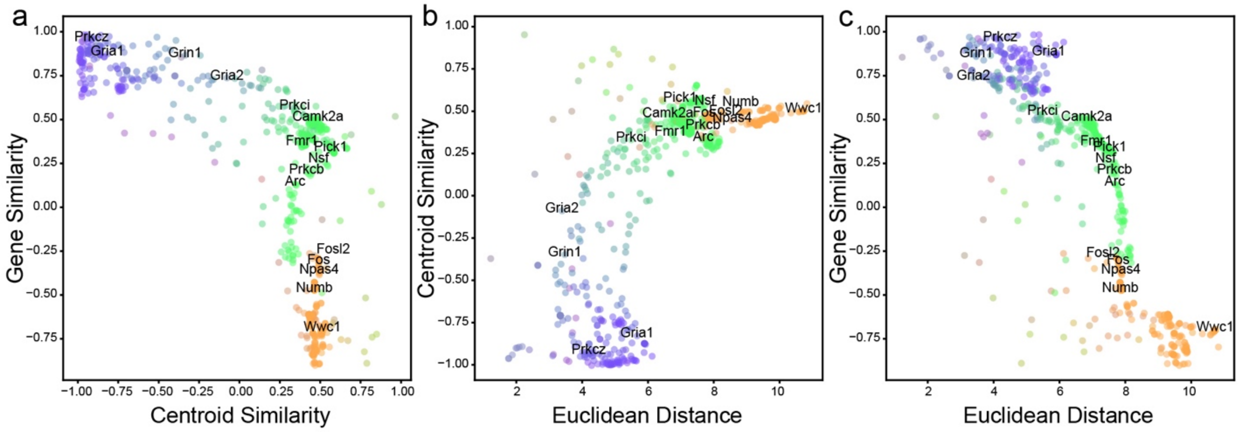

Figure S13. Two-dimensional scatter plots of selected metric pairs. These plots sufficiently illustrate the co-clustering trends observed in the data without requiring dimensionality reduction methods such as t-SNE or UMAP. The same RGB color map is used as for the main training clusters.

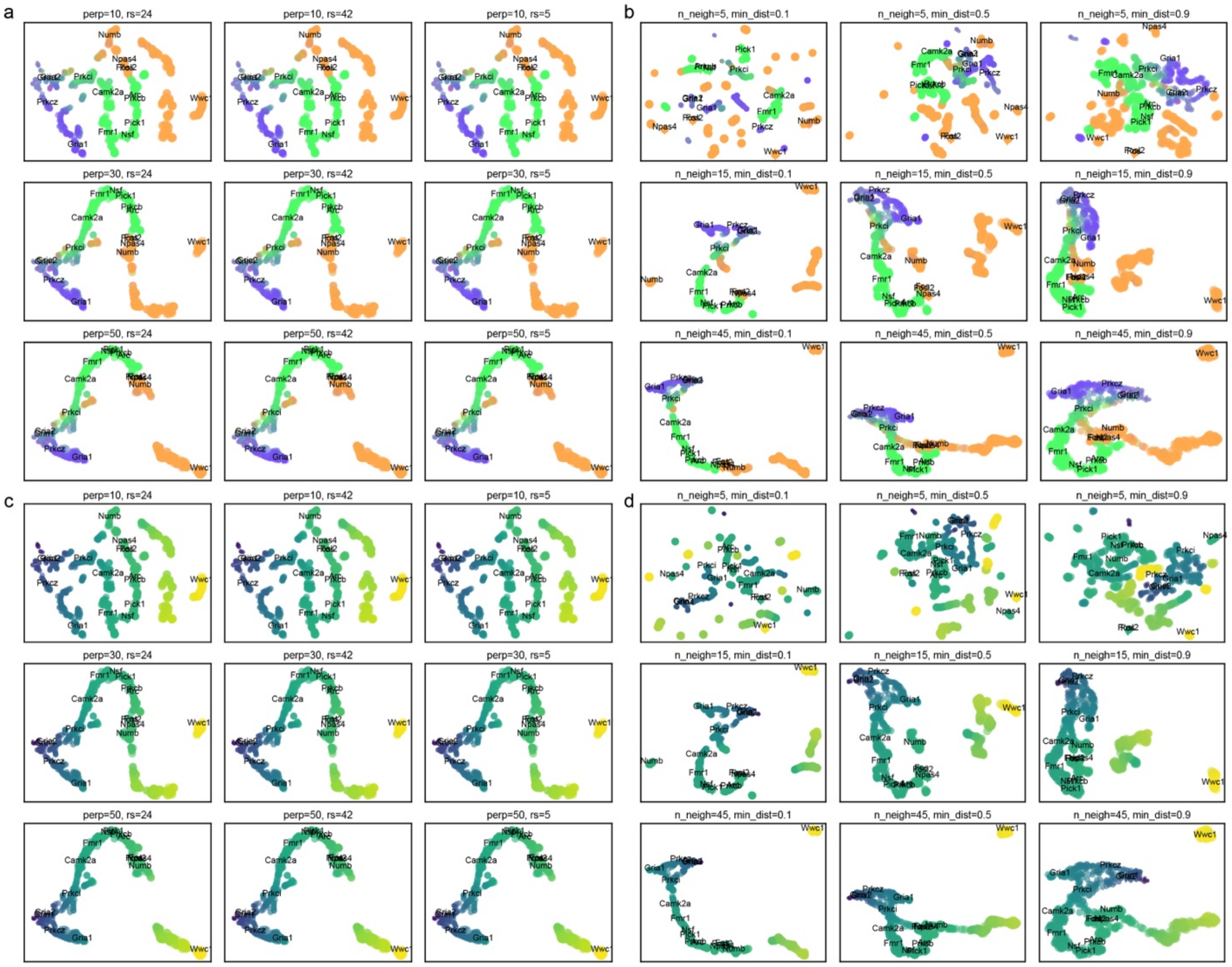

Figure S14. Robustness of t-SNE and UMAP Dimensionality Reductions Across Parameter Settings.
a) and c) t-SNE embeddings generated using varied perplexity (perp) and random seed (rs) settings. b) and d) UMAP embeddings generated using number of neighbors(n_neighbor) and minimum distances (min_dist). We used two coloring methods: 1) RGB color utilizing PC1, PC2 and PC3 (a, b) and 2) the color of the magnitude of Euclidean Distance changes from the yoked to the trained data set (c, d).

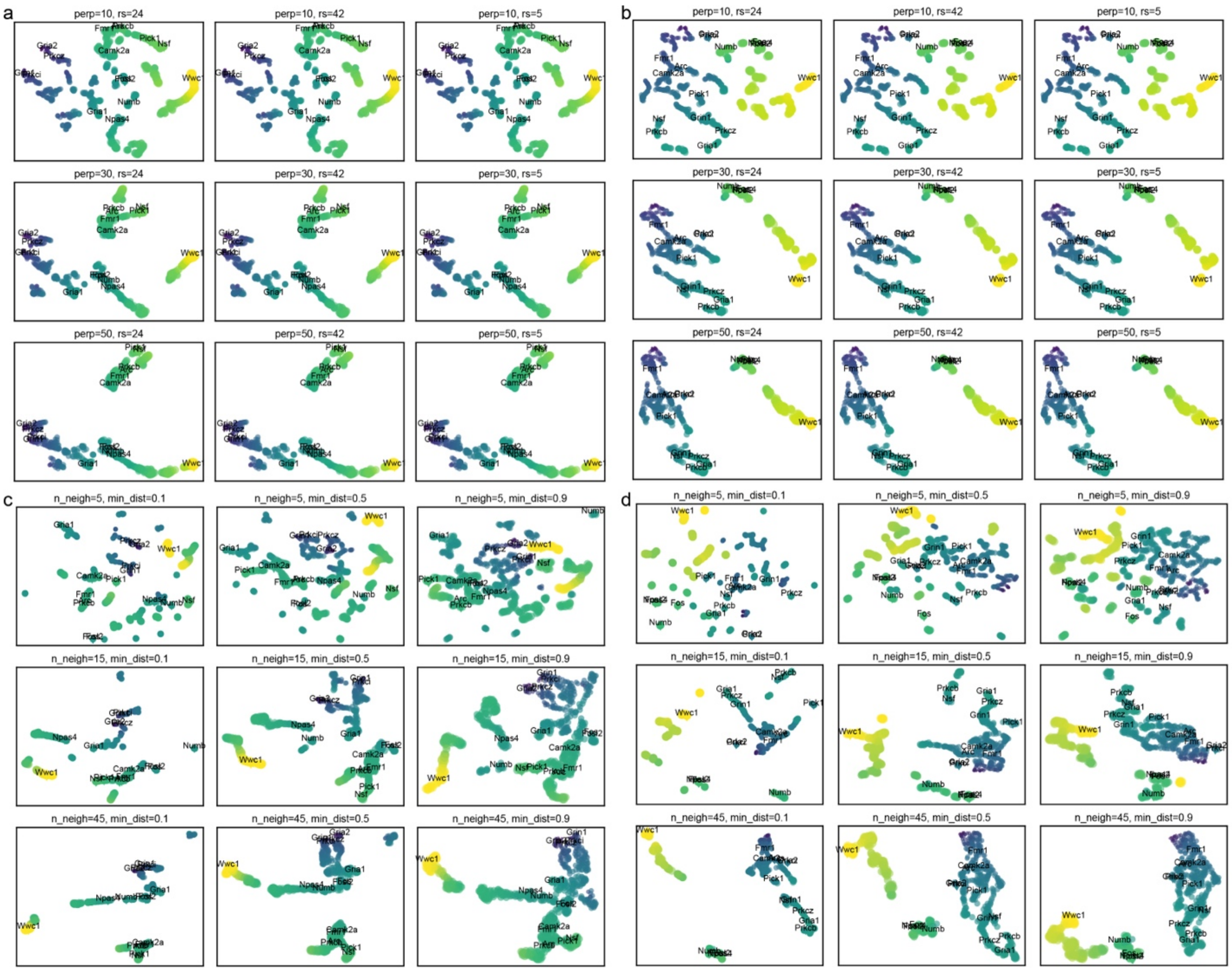

Figure S15. Ablation study using different gene-gene correlation measurements.
To test the robustness of the correlation method selection, we changed the gene-gene correlation measurements to the absolute value of Spearman correlation (abs(Spearman)) and Xi-Correlation (Xicor). The parameters varied are the same as the Figure S11. a) and c) are t-SNE and UMAP embedding results using abs(Spearman). b) and d) are results in the same order but using Xicor. The color represents the magnitude of Euclidean Distance from yoked to trained samples.
